# The Carbon Cost of Travel to a Medical Conference: Modelling the Annual Meeting of the Canadian Association of Gastroenterology

**DOI:** 10.1093/jcag/gwab021

**Published:** 2021-07-29

**Authors:** Desmond Leddin, Ciaran Galts, Elizabeth McRobert, John Igoe, Harminder Singh, Paul Sinclair

**Affiliations:** Department of Medicine, Dalhousie University, Halifax, Nova Scotia, Canada; University of British Columbia, Vancouver, British Columbia, Canada; Dalhousie University, Halifax, Nova Scotia, Canada; Department of Medicine, University of Manitoba, Winnipeg, Manitoba, Canada; INSINC Consulting Inc., Toronto, Ontario, Canada

**Keywords:** Canada, Carbon cost, Climate change, Gastroenterology, Medical conference

## Abstract

**Objectives:**

We estimated and compared the travel related carbon emissions of the annual meeting of the Canadian Association of Gastroenterology between the two most common geographical locations of the meeting.

**Methods:**

We modelled the car, train and flight travel journey of each registrant to two annual meetings. One was held in Toronto, close to where the majority of Gastroenterogists live, the other in Banff in the west of the country. We used validated carbon emission outputs per kilometer of travel.

**Results:**

The average per capita distance travelled to the Toronto meeting was 2845 km, resulting in 0.540 tonnes (t) of CO_2_equivalent (CO_2_e) emitted per person. When the meeting was held in Banff emissions were 41% higher than those in Toronto with an average distance travelled of 3949 km and 0.760t of CO_2_e emitted per person. Almost all of the travel related carbon emissions for both meetings were generated by flying.

**Conclusions:**

Even when held close to the largest population centre, there is a large environmental impact from travel to annual meetings. Importantly, choice of meeting location has a very big impact on difference in carbon emissions. Societies need to consider the site of meetings and reduce the number of in-person attendees if they wish to reduce their carbon footprint. Hybrid models participants should be considered. Our analysis also suggests, other medical societies who wish to model their annual meetings can use a simplified model, using flying distance only, to estimate travel-related emissions.

## Introduction

Climate change has been described as the greatest public health threat of the 21st century (1). It has major implications world-wide and for all aspects of personal and professional lives including those involved in digestive health and disease (2).

It is primarily caused by rising atmospheric greenhouse gas (GHG) concentrations as a result of combustion of fossil fuels. There are many different GHG such as CO2, methane and nitrous oxide. Each has a different atmospheric warming potential and duration of effect. CO_2_ is the most abundant GHG, and the others are referenced regarding CO_2_ as the standard. When multiple GHG are released, for example, by jet flight, the total impact of the gases released is summarized as CO_2_ equivalents (CO_2_e). Travel, with its associated carbon emissions from driving and flight, is a significant contributor to GHG emissions (3).

Health care providers and scientists have a role to play in mitigating the effects of climate change through education, advocacy, and changes in personal and professional behavior, including travel which can make up a significant portion of an individual’s annual carbon footprint (4).

The Canadian Association of Gastroenterology (CAG) consists of over 1000 members, health care providers and scientists, who are involved in digestive disease and health. An annual meeting, Canadian Digestive Disease Week (CDDW), is held for the purposes of promoting the objectives of the Association. Most of the membership (approximately two thirds), live in the Provinces of Ontario and Quebec (5), which are in the east-central area of the country. Sixteen per cent of the membership is located in Alberta. The location of the annual CDDW meeting varies, typically alternating every other year in a western location (often in the resort town of Banff) or central-eastern location, Toronto or Montreal. Canada measures over 6500 km from the east to the west coast. Therefore, regardless of where CDDW is held, some of the membership must travel considerable distances to attend the meeting.

The objectives of this study were twofold. Firstly, to determine the travel related carbon emissions of the annual CDDW meeting. Secondly to compare the magnitude of the effect of holding the meeting close to where most gastroenterologists live (Toronto) to that in a more scenic site (Banff, Alberta). The last two meetings have been held in these two locations. Our hypothesis was that holding the meeting in a location closer to where most members of the CAG live, namely Toronto in the east-central zone, is associated with reduced travel related carbon emissions, but were surprised at the magnitude of the effect we found.

## METHODS

### Postal Codes

The Canadian postal code is alphanumeric and consists of six digits, for example, B3H 2L0. The first digit (letter) denotes a Province with the exception of the Provinces of Quebec (which has three codes) and Ontario (which has five codes). There is a specific digit for the cities of Toronto (M) and Montreal (H). The second digit (number) represents a specific rural region, an entire medium-sized city, or a section of a major metropolitan area. The third digit (letter) represents the postal district in the rural region/city. For example, the code B3H represents the Province of Nova Scotia (B), the city of Halifax (3) and the postal district within Halifax (H). The last three digits of the postal code allow location of a street address. In order to preserve confidentiality, we did not review the data for these final three digits.

### Ethics

We consulted with the Capital Health ethics board in Halifax. The study falls under Tri Council Policy Statement section 2.4 on secondary use of anonymous information. It did not require a research ethics board review.

### Inclusion

We obtained the data from the Canadian Association of Gastroenterology on the attendees at the last two in-person meetings. The inclusion criteria included the following:

Attended CDDW in Toronto (2018) or Banff (2019)
Canadian postal code available
Member (Regular or Trainee of the CAG)

### Exclusion

International participant
Industry/sponsor participant

### Travel Distance Model

We determined the originating site of travel, from the first three letters of the postal code of residence, of attendees at the Toronto and Banff Meetings.

The total distance for each person was calculated from three components.

A. Distance to departure: If the attendees needed to fly to the conference and there was an airport within 50 km of the residence, we discounted this distance because we could not accurately determine the point of departure. If the attendee needed to drive, or take a train, more than 50 km to the airport the round-trip distance to the departure airport was calculated. If the attendee did not need to fly, no distance charge to an airport was added.
B. Flight distance. We calculated the distance from the departure airport, if one was likely used, to the arrival airport in Toronto or Calgary (the airport for Banff).
C. Finally, we added the travel distance from Calgary airport to Banff; a round trip of 240 km. The conference centre in Toronto is located in the centre of the city, 30 km from the airport. We did not add this distance to the travel distance.

### Adjustments for the Banff Meeting

Calgary is the major airport for Banff and also has an academic centre. There is a second major urban area in Alberta, Edmonton, 300 km north of Calgary which also has a medical faculty. We assumed that all of the Gastroenterologists in the two major urban areas of Alberta drove to the meeting, that they drove alone, and used a medium-sized car. We assumed that all attendees from outside Alberta flew to Calgary and then travelled the 240-km round trip by coach to Banff.

### Adjustments for the Toronto Meeting

Participants who lived within the metro Toronto area (postal code starting with M), were not given a mileage charge to the Toronto meeting.

There are nine academic gastroenterology centres, with significant numbers of CAG members, within potential driving, or train, travel distance to Toronto. All, with the exception of McMaster, have flight connections to Toronto. Attendees from these centres have a choice of travel modes which include road, rail and air. Based on input from educators at these centres, we modelled travel patterns (Table 1) from the cities with these academic centres.

**Table 1.** Major urban centres round trip distance to Toronto in kilometers, and the percentage of attendees from each city travelling by car, train or air

City (Academic centre)	Round trip to Toronto (km)	Drive (%)	Train (%)	Fly (%)
Quebec	1560	5	15	80
Sherbrooke	1402	20	20	60*
Montreal	1100	20	20	60
Ottawa	900	20	20	60
Kingston	526	20	80	0
London	384	30	50	20
McMaster	140	90	10	0

*Attendees from Sherbrooke are assumed to drive to Montreal if flying or taking the train, so an additional 300 road kilometers is added to their journey.

### Measurement of Distance

We used Google maps to calculate driving distance, Via Rail comparison tool to determine train distance, and airport.globefeed.com/Canada (6) to measure flight distance. Flight distance was estimated by taking the geographic flight distance from the nearest airport to Toronto or Calgary. We did not include extra travel for connections since we do not know if attendees might have taken a direct or connecting flight.

### Calculation of Carbon Cost Per Kilometer

The carbon cost per kilometer of travel varies with mode of travel. Burning of fossil fuels generates several gases, each of which has a different warming potential. In order to allow comparison, the travel carbon cost per kilometer is expressed in CO_2_equivalents (CO_2_e). We used the following estimates. Short-haul flights were defined as those less than 1500 km one-way, long haul were greater than 1500 one way (7).

We used the carbon emissions per kilometer of travel listed in Table 2.

**Table 2.** CO_2_ equivalents in grams per kilometer per person for car, train, coach and air travel

Mode of transport	g CO_2_e/km/person
Car, medium sized	170
Train	41
Coach	27
Short-haul flight <1500 km	158 Average
	155 Economy
	233 Business
Long-haul flight 1500–8000 km	195 Average
	149 Economy
	434 Business

Flight emissions are shown for short and long haul, average passenger, economy class and business class.

Estimates were taken from UK Government Department for Business, Energy and Development Conversion factors 2019 (8).

## RESULTS

The numbers of attendees from each of the 10 Provinces are given in Table 3. There were no attendees from the Territories. Attendee numbers were 6% higher in Toronto than Banff. The percentage of trainees (48% in Toronto, 51% in Banff) and Provincial representation were similar at both meetings. A comparison of the Banff and Toronto meetings is shown in Table 4. The two largest blocks of attendees, as a percentage of total attendees, for both the Toronto and Banff meetings respectively, were from Ontario (33%, 37%) and Alberta (26%, 26%).

**Table 3.** Total number of trainees + members, by Province attending the Toronto and Banff meetings

	NL	NS	NB	QC	ON	MB	SK	AB	BC	Total
Toronto 2018	8	22	6	82	177	28	8	140	63	534
Banff 2019	5	18	3	81	189	16	8	129	53	502

AB, Alberta; BC, British Columbia; MB, Manitoba; NB, New Brunswick; NL, Newfoundland and Labrador; NS, Nova Scotia; ON, Ontario; QC, Quebec; SK, Saskatchewan.

**Table 4.** A comparison of the Toronto and Banff meetings with regard to the number of registrants (N), distance travelled in kilometers (km) and CO_2_e generated round trip, flying, driving and train travel

	Toronto *N* = 534		Banff *N* = 502	
Travel segment	Distance, km	CO_2_e tonnes	Distance, km	CO_2_e tonnes
Departure Airport	15,734	2.7	20,528	3.5
Flight - Short Haul	131,348	20.7	123,874	19.6
Flight - Long Haul	1,322,642	257.9	1,708,864	333.2
Flights - Total	1,453,990	278.6	1,832,864	352.8
Driving	36,872	6.3	149,770	25.5
Train	28,432	1.2	0	0
Total Travel	1,519,249	288.8	1,982,634	381.8
Average per person	2845	0.540	3949	0.760

Carbon is expressed in metric tonnes (t), one tonne equals 1000 kg. Average values of CO_2_e were used for flight emissions.

The total distance travelled by registrants to the Toronto meeting was over 1.5 million km with an average of 2845 km per attendee. Travel to the departure airport was a very small proportion (1.0%) of total travel. The travel distance of attendees who drove to the Toronto meeting was 2.4% of all travel, and train travel was 1.9%. Ninety-six per cent of travel to the Toronto meeting was flight related, 91% of this was related to long-haul flights in excess of 1500 km per flight segment. With regard to carbon emissions, 96% of the emissions were related to flying. The total CO_2_e generated was 288.8t of carbon dioxide. This equates to 0.540t of carbon dioxide per attendee at the meeting.

The total distance travelled by attendees at the Banff meeting was nearly 2 million km, averaging nearly 4000 km per attendee. Travel to reach the departure airport was 1% of total, similar to the Toronto meeting. The travel distance of attendees who took a car or coach to the Banff meeting was more than the Toronto meeting at 7.6% of all travel. This reflects both travel from Edmonton and the return coach journey to Banff of arrivals at the airport in Calgary, which generated 4.4% of all travel kilometres. There is no effective train connection between Calgary, Edmonton and Banff, so train travel was not a factor. Over 92% of travel to the Banff meeting was flight related, 93% of this was related to long-haul flights. With regard to carbon emissions 92.4% of the emissions were related to flights. The total CO_2_e was over 380t, which is equivalent to 0.760t of CO_2_e per attendee.

The average distance travelled to the Banff meeting was 1104 km more than that of the Toronto meeting and the CO_2_e emissions 220 kg higher per person.

### Contribution of Road and Rail Travel

The estimated travel distances to the Toronto meeting by attendees from parts of Quebec and Ontario was based on expert opinion on the percentages who might have travelled by road, rail or air. Using the expert opinion model, the distance flown was 61,079 km by Ontario and Quebec attendees. In a sensitivity analysis, we estimated the flight travel distance if all of these travelled by air as opposed to travelling by a mixture of road, rail and air. If all attendees from Ontario and Quebec flew, we estimated the flight distance at 113,876 km, a difference of 52,797 km. from the expert opinion model. Given the total distance traveled to the Toronto meeting was 1,519,294 km, the difference between expert opinion and possible all flight model is 3% and not likely a major source of error.

We did not add a travel cost for attendees from the Toronto area, postal code M, to the Toronto meeting as we did not know their departure point. The maximal distance within this area to the convention centre is 50 km return. There were 60 registrants from postal code M. If we assigned a 50 km travel distance to each, the total travel contribution would be 3000 km daily, or 9,000 km for the 3-day meeting, an insignificant contribution given the total meeting travel of over 1.5 million kilometers.

### Contribution of Air Travel

Air travel represented 92% of travel to the Banff meeting and 96% of travel to the Toronto meeting. We chose carbon emission values of 158g CO_2_e/km for short-haul flights and 195 g CO_2_e for long-haul flights. These values represent the average passenger. Short haul was defined as <1500 km, long haul >1500 km. However, it is likely that some attendees flew business class. As shown in Table 5, the flight related emissions vary by flight class.

**Table 5.** The effect of travel class on short haul, long haul and total CO_2_e emissions to the Toronto meeting

	Short haul Emissions g CO_2_e/km Total CO_2_e	Long haul Emissions g CO_2_e/km Total CO_2_e	Total CO_2_e emissions	Total CO_2_e as a per cent of average
Average	158 20.7t	195 257.9t	278.6t	
Economy	155 20.3t	149 197.1t	217.4t	−22%
Business	233 30.6t	434 574.0t	604.6t	+117%

Emissions per kilometer by flight class and subsequent total emissions are shown for the average passenger, economy and business class.

If all attendees flew in economy, emissions would be reduced by 22%. If all attendees travelled in business class, the flight-related emissions would more than double. We do not know what percentage of attendees flew in economy or business.

## Discussion

When the annual CAG meeting was held in the vicinity of where most attendees live, the Toronto – Montreal axis, we estimated the travel related carbon emission total was 288t, nearly 540 kg of CO_2_e per attendee. Emissions were estimated to be 41% higher when the meeting was held distant from the largest Canadian population centres. Thus, choice of meeting location is extremely important with regards to the carbon footprint of the annual society meetings. Meeting organizers should consider this when choosing a venue. We are not aware of any other estimates of environmental impact of Canadian medical meetings and suggest all societies should include such estimates when planning their meetings.

Regardless of where the meeting is held it will be associated with significant emissions. One kg of CO_2_ is equivalent to 600 L of CO_2_ gas, therefore each attendee at the Banff meeting results in nearly a half million liters of travel related CO_2_ being released into the atmosphere where it persists indefinitely and will contribute to warming of the atmosphere for millennia. The average distance driven of a car in Canada is 20,000 km per year with a fuel efficiency of 9 L/100 km. The annual fuel consumption will be 1800 L of gasoline (9). This will generate 4320 kg of CO_2_e. Therefore, the travel related CO_2_ emissions of each attendee at the annual meeting of the CAG in Banff, 760 kg, is equivalent to driving a car for about two months. Taking a global perspective, the average carbon emissions per person globally are about 5t per year (10). Each attendee at the Banff meeting generates in travel alone, for a meeting of a few days, 2 months of the carbon emissions of the average human.

We did not include industry or sponsor attendees, international speakers, spouses or partners, nor did we include the carbon cost of the convention centre, disposables, audiovisual, printing, hotel stay or, in the case of industry, shipping booths and equipment. The actual carbon cost of the annual meeting is very much higher than the travel emissions which we are reporting. Most of the patient advocacy groups and most industry companies are located in Quebec and Ontario. If we included these in the calculations, the increased cost of the Banff meeting would be even higher.

Our estimates are dependent on the carbon cost of flying. Measuring the CO_2_e emissions of flying is complex. It depends on distance flown, type of plane, economy or business class seat, and also whether an effect known as radiative forcing is taken into account. Radiative forcing factors in the additional environmental impact of aviation such as the high-altitude release of water vapor and nitrous oxide (11). We do not know what proportion of attendees flew in economy or business class, which as shown in Table 5 could be an important determinant of emissions. A more accurate estimate of travel related carbon could be obtained with detailed data on the travel of attendees, and agreement on the carbon cost per kilometer travelled, but our data provide reasonable estimates and are in keeping with those reported previously (12–14).

We used Canadian postal codes to model quite precise departure points and travel routes but, in retrospect, this was unnecessary; all that is required is knowledge of the return flight journey distance. Other societies which may wish to model the travel related impact of their meeting can likely simply measure flight distances of their attendees. This may only apply to meetings which are national or international in scope, in countries with geography similar to Canada’s, and where the association members are primarily urban based.

Meetings serve important functions with regards to administration, research, education and networking and they may, also, have a role to play in professional identity formation which is achieved in part through socialization (15). Objective evidence on how effectively medical conferences meet these goals is lacking and concerns have been expressed about their usefulness (16–18).

However, given their popularity, it is clear that they meet a need. The question then becomes how to conduct meetings which meet the objectives of the Association and the needs of members while minimizing the environmental impact?

Holding the meeting close to where most people practice, Toronto, will reduce flight kilometers, as will decreasing the number of attendees. We did not model the emissions for a meeting in Montreal (the second largest population center in Canada), but they are likely to be similar to Toronto. With regard to decreasing attendance, we need to understand which objectives are best served by face-to-face meetings and which can be met online. For example, nearly half of the attendees were trainees. It may be that the benefits of bringing members of the profession together at earlier stages of their career to encourage networking and build professional identity outweigh the negative environmental impact. Conversely, there may be some members of the profession who do not need to attend in person. Future needs analysis needs to include the traditional educational objectives as well as other domains, such as professional development, and networking, which are not always explicit.

In addition to modifying the location of the meeting and who needs to attend in person, there are other options to the traditional format. These include holding a simultaneous western and central meeting perhaps with faculty representatives at both sites. The difference in travel emissions between the meetings in Banff and Toronto may be less than expected for some readers. The explanation appears to be that when the meeting is held in Ontario, the second largest block of gastroenterologists (those from Alberta, 26% of attendees) fly to Toronto and when the meeting is held in Alberta the largest block of gastroenterologists (those from Ontario, 38% of attendees) fly to Calgary. Moreover, the number of attendees from Ontario and Quebec are almost equal to those from prairies/western provinces (B.C., M.B., S.K., and A.B.). Given that the round-trip distance from Calgary to Toronto is 5400 km, it is inevitable that a single meeting will generate considerable carbon emissions. A two-site concurrent meeting would provide an in-person meeting for the majority of Gastroenterologists while minimizing long-haul flights. Canada has a large geographical distribution and hence innovative travel, and meetings options need to be considered to reduce our carbon footprint. France has recently banned short domestic flights, where the alternative is 2.5 hours or less by train. Such an initiative may have limited impact in a large country such as ours.

A biennial meeting would reduce emissions by 50% with no change of venue. Virtual meetings, and hybrid meetings where some attend in person, may also have a role (19, 20). Emissions from virtual conferences are negligible compared to in-person events and are primarily due to network-related energy use (21).

A reduced number of attendees may make the traditional attendance of industry at medical conferences cost ineffective. Clearly, this requires discussion and planning with industry supporters. Alternative virtual presence or distributed numbers at two sites should be considered.

If in-person meetings are required, then there are guides which will help reduce the carbon footprint and steps which can easily be taken by meeting planners. These include planning for waste management, changes to catering and choosing venues which are energy and design environmentally friendly (21, 22). Encouraging attendees to avoid business class travel will also reduce emissions. Carbon offsetting can also be considered. One initial essential step which all medical conference planners should take would be to incorporate carbon reduction into the organization of the meetings.

The COVID pandemic has shown that it is possible to continue the activities of medical societies online. Given the emerging climate crisis, we believe that societies should look at their annual conferences, and other programs which involve travel, not only from a financial cost-benefit perspective but also with regard to the environmental cost. The reduction in Canada’s annual GHG emissions of 729 million tonnes (23) by moving to a net carbon zero CAG annual meeting would be insignificant at less than 0.5 × 10^-6^ of annual national emissions. The impact of the leadership shown on this health emergency, and the message sent with regard to the climate crisis, to attendees and all other partners in the annual meeting is likely to be far more impactful.
